# Does an educational intervention improve parents’ knowledge about immunization? Experience from Malaysia

**DOI:** 10.1186/1471-2431-14-254

**Published:** 2014-10-06

**Authors:** Ammar Ihsan Awadh, Mohamed Azmi Hassali, Omer Qutaiba Al-lela, Siti Halimah Bux, Ramadan M Elkalmi, Hazrina Hadi

**Affiliations:** Department of Pharmacy Practice, Kulliyyah of Pharmacy, International Islamic University Malaysia, 25200 Kuantan, Malaysia; Discipline of Social and Administrative Pharmacy, School of Pharmaceutical Sciences, Universiti Sains Malaysia, 11800 Penang, Malaysia; School of Pharmacy, Faculty of Medical sciences, University of Duhok (UOD), Duhok, Iraq; Department of Pharmaceutical Technology, Kulliyyah of Pharmacy, International Islamic University Malaysia, 25200 Kuantan, Malaysia

**Keywords:** Immunization, Parents, Educational intervention, Knowledge, Malaysia

## Abstract

**Background:**

Parents’ knowledge about immunization is an important predictor factor for their children’s immunization status. The aims of this study were to assess parents’ knowledge and to evaluate the effect of a short educational intervention on improving parents’ knowledge of childhood immunization.

**Methods:**

A cross-sectional study using a pre- and post-test intervention survey of a single group was conducted among Malaysian parents. Changes in total knowledge score before and after the intervention were measured using a validated questionnaire. The intervention consisted of an animated movie and lecture using simple understandable language. Wilcoxon signed ranks test and the McNemar *x2* test were applied to compare the differences in knowledge before and after the intervention.

**Results:**

Seventy-three parents were enrolled in this study; the majority were mothers (n = 64, 87.7%). Parents’ knowledge about childhood immunization increased significantly after the intervention compared to the baseline results (*p* < 0.001). There were significant differences between parents’ knowledge and their educational level and monthly income (*p* < 0.001 and *p* = 0.005), respectively.

**Conclusions:**

A short educational intervention designed for parents had a positive effect on their knowledge about immunization. Educational interventions targeting parents with low levels of education and income are needed. Further studies investigating the actual effectiveness of such interventions on immunization rates and statuses are required.

## Background

In recent years, vaccine hesitancy has been the subject of growing attention as an emerging term in the literature [1, 2]. Vaccine-hesitant individuals have been defined as “a heterogeneous group in the middle of a continuum ranging from total acceptors to complete refusers” [3]. The three key determinants of vaccine hesitancy are: contextual influences, individual and group influences (including knowledge and awareness), and vaccine and vaccination-specific issues [3, 4]. The global substantial reduction in the prevalence of vaccine preventable diseases makes parents have little or no experience with such diseases, and as a result, the benefits of vaccination and the risks of not vaccinating are not appreciated as much as they were in 20th century.

The success of childhood vaccination has made seeing a child with measles or polio very rare. However, there are constantly reports in the media and internet about adverse vaccine reactions and concerns about the safety of vaccines. Parents’ decisions can be negatively influenced by the huge amount of conflicting vaccine-safety information and misinformation on the internet [5, 6].

Factors related to immunization services and parental knowledge and attitudes were the main reasons for incomplete or no vaccinations. In a review of 126 documents of the grey literature to identify reasons why eligible children had incomplete or no vaccinations, lack of parental knowledge was the most cited factor in 58 of the documents [7]. Many studies have found that parents’ lack of knowledge about vaccines is a problem that leads to low vaccination coverage [8–13]. It has been found that children of mothers who have knowledge about immunization and its importance had much greater immunization rates compared to children whose mothers did not have immunization knowledge [14]. Caregivers who vaccinated their children on time had higher vaccine related knowledge than those who delayed [15]. Parents’ knowledge about vaccine schedules is a predictor factor for children’s immunization status [16, 17]. The negative attitudes among parents are mainly due to a lack of knowledge about the importance and safety of vaccines [8].

According to the Department of Public Health, Malaysia has high immunization coverage ≥95 [18]. The continued success of the Expanded Programme of Immunization (EPI) in Malaysia relies on high immunization coverage, which in turn requires parental understanding of the importance of vaccination and the willingness to vaccinate children. Parents’ lack of knowledge about the timing of the immunization schedule was a significant predictor of incomplete immunization in Malaysia [19].

Recently, there has been a paradigm shift from efforts to increase not just immunization coverage but also to improve immunization timeliness. Around the world, more attention and understanding by health care professionals about the health and well-being of young children is dependent on parents (especially mothers) who understand the importance of immunization and follow the recommended immunization schedule [20–22]. Taking into account the importance of both parents and health care workers in decision making, educational strategies to increase their knowledge in the area of vaccine safety systems might change beliefs and improve trust in the system [23]. Attention should be given to mothers with incorrect knowledge and poor perception of immunization; moreover, it is important to consider that in the immunization programmes [24].

Educating mothers whose children are at risk of not completing the immunization schedule is an important strategy to improve immunization coverage [25]. An educational programme about the importance of immunization is needed, especially for parents with a lower educational level, in order to improve the immunization rate [26–29]. To our knowledge, no study in Malaysia has assessed the impact of educational programming for improving Malaysian parents’ knowledge of their children’s immunization.

### Study objectives

The objectives of this study were to assess the knowledge of Malaysian parents about childhood immunization and to evaluate the effectiveness of an educational seminar for improving parents’ knowledge about childhood immunization, and to compare parents’ knowledge scores across select demographic characteristics.

## Methods

### Study site and research design

This study was conducted in Kuantan, the state capital of Pahang, the largest state in Peninsular Malaysia, with an area of 2,960 km^2^ and population of 450,211 (2010 census). Parents who were attending the Health Clinic Indera Mahkota, which provides maternal and child health services to Malaysian citizens, were invited to participate in an educational seminar on immunization. This study utilised a one group pre-test – post-test design to assess the impact of an educational seminar among Malaysian parents. Seventy-three fathers and mothers agreed to participate in this educational seminar. The original educational session content was prepared in English by the experts from the School of Pharmacy and was translated into the Malaysian language (Bahasa Melayu) and delivered via a pharmacist with expertise in the field of immunization. In order to get the baseline knowledge about immunization, pre-evaluation questionnaires were administered to parents who attended the seminar. A post evaluation after the educational seminar was conducted and aimed to evaluate parents’ knowledge towards immunization and the impact of the intervention.

### Recruitment and enrolment

Before the study began, the researchers met with the medical officer in charge and nurses in the clinic and provided them with a detailed description of the purpose of the study. Parents who were visiting the clinic for any reason, had a child younger than two years old, and lived in Kuantan were invited to attend the seminar. Parents who did not have a child younger than two years old and lived outside Kuantan were excluded. Two posters with information about the seminar were displayed in the clinic for a two-week period. The nurses in the clinic gave more details and an explanation of the study to eligible parents as well as a brochure explaining the purpose and content of the seminar. Parents who were interested in attending the seminar were asked to register and to attend the seminar on the proposed date.

### Intervention

The educational seminar was designed for parents in simple understandable language; the educational materials were adapted from available sources such as the Centres for Disease Control and Prevention (CDC) and were translated into the Malaysian language (Bahasa Melayu). The content of the educational materials was prepared in order to include issues on the importance of immunization, immunization type, immunization schedule, side effects and contraindications, and immunization doses. Face and content validity of the material after translation was made by three Malaysian pharmacists who are experts in the field, and modifications were made to suit the culture and context of Malaysia. The seminar was delivered through an educational animated movie (10 minutes) and a didactic lecture using a PowerPoint slide presentation (50 minutes). At the end of the seminar, the platform was open to parents to ask questions and get their feedback and concerns. The parents were expected to gain better knowledge about immunization in order to increase the immunization rate and maintain their child’s/children’s immunization status.

The key learning outcomes of the seminar included: understanding the importance of immunization as an important way to protect the children and society in general from the vaccine preventable disease, highlighting the diseases that can be prevented by vaccines, the role of the parents (as they are the decision maker for their children) and to weigh the facts of disease side effects and vaccine side effects and the importance of getting the vaccine at the right time.

### Survey instrument

To achieve the objective of the study, a questionnaire in the Malaysian language (Bahasa Melayu) designed mainly to assess parents’ knowledge about immunization was used. The questionnaire was validated by three specialist pharmacists, who are experts in this field, and then the questionnaire was piloted among 88 Malaysian parents. Reliability was assessed by internal consistency of the questionnaire reporting Cronbach’s alpha coefficient of 0.757.

The questionnaire consisted of two parts: (I) sociodemographic characteristics of the parents such as gender, age, race, religion, marital status, place of living, number of pre-school children, family size, employment status, educational level, and family income and (II) structured items concerning basic knowledge about immunization (10 questions). The questions consisted of closed-ended questions (yes/no).

### Data collection

Before becoming involved in the study, all parents who agreed to attend the seminar were given a cover letter describing the study objectives and time needed to complete the questionnaire as well as a written informed consent form. The educational seminar was delivered to the parents who were registered and attended the seminar. One group pre-test/post-test survey was conducted and the differences in the scores on knowledge before and after the seminar were measured. Ethical approval for the study was obtained from the Medical Research Ethic Committee (MREC) and the National Institutes of Health (NIH), Ministry of Health Malaysia (Registration ID: NMRR-13-485-15673). Participation was voluntary and the responses were anonymous. The evaluation session was held at the seminar room at the Health Clinic Indera Mahkota in Kuantan in September 2013. The seminar was planned over one hour and a seminar manual containing relevant material was prepared. All parents were asked to complete a questionnaire regarding sociodemographic characteristics and knowledge about immunization before and after the educational seminar.

### Statistical analysis

SPSS version 20.0 software package (SPSS Inc., Chicago, IL, USA) was used to analyse the data. Both descriptive and inferential statistics were used whenever appropriate. Frequency and percentage of each demographic data parameter, namely gender, race, religion, place of living, and age, were determined. Next, family data, including marital status, number of pre-school children, family size, employment status, educational level, and family income, were also evaluated.

The percentages and frequencies of parents’ demographic data (categorical variables) were evaluated, and means and standard deviations were calculated for knowledge scores (continuous variables). Scoring of the questions was determined by giving one point (1) for each correct answer and zero (0) for incorrect answers or no response (don’t know).

Mean and median scores for each parent and statement were calculated. The maximum possible score was 10, in the case that the respondents chose all the correct answers for each statement. Kruskal-Wallis and Mann–Whitney tests were used to determine the differences among groups pre and post seminar for non-parametric distributions. The Wilcoxon signed ranks test was used for continuous data, and the McNemar *x2* test was used for categorical data to compare the differences in knowledge before and after the educational programme for non-parametric distributions. A *p*-value of 0.05 or less was considered to be significant.

## Results

### Sociodemographics characteristics

A total of 73 parents were registered and attended the seminar. The majority of the parents were mothers (n = 64, 87.7%); nine (12.3%) were fathers. Around half of the parents were between 30 and 40 years old (n = 35, 47.9%). Most of the participants were living in an urban area and employed (n = 66, 90.4% and n = 59, 80.8% respectively). The characteristics of the parents are summarised in Table 1.

**Table 1:** **Socio-Demographic Characteristics of the participants (N = 73)**

Characteristics	Frequency	(%)
**Gender**		
Male	9	(12.3)
Female	64	(87.7)
**Age**		
20-30	30	(41.1)
30-40	35	(47.9)
>40	8	(11.0)
**Marital Status**		
Married	67	(91.8)
Single	6	(8.2)
**No. of Preschool Children**		
1-2	61	(83.6)
3-4	12	(16.4)
**Family Size**		
<4	44	(60.3)
4-6	24	(32.9)
>6	5	(6.8)
**Race**		
Malay	66	(90.4)
Chinese	7	(9.6)
**Religion**		
Islam	66	(90.4)
Buddhism	7	(9.6)
**Place of Living**		
Rural	7	(9.6)
Urban	66	(90.4)
**Employment Status**		
Employed	59	(80.8)
Unemployed	14	(19.2)
**Education Level**		
Primary	9	(12.3)
Secondary	21	(28.8)
Tertiary Edu	43	(58.9)
**Family Income**		
< RM 1000	5	(6.8)
RM 1001-2000	18	(24.7)
RM 2001-3000	15	(20.5)
RM 3001-4000	16	(21.9)
>RM 4000	19	(26.0)

### Parents’ knowledge scores

The pre-test and post-test results for the individual items in the knowledge assessment are presented in Table 2. The number of parents answering correctly increased on all of the ten items. Of these ten items, the pre- and post-test scores for seven items were significantly different: (1) Healthy children do not need immunization (78.1% vs. 94.5%; *p* = 0.002); (2) Vaccination is for all ages (52.1% vs. 79.5%; *p* < 0.001); (3) In some health situations, vaccines should not be given (58.9% vs. 82.2%; *p* < 0.001); and (4) Vaccines can be given in combination (67.1% vs. 93.2%; *p* <0.001).

**Table 2:** **Comparison of parents’ knowledge about immunization before and after the educational intervention (N =73)**

Questions	Correct response before intervention n (%)	Correct response after intervention n (%)	***P***value
Healthy children do not need immunization	57 (78.1)	69 (94.5)	0.002
There are different types of vaccines	60 (82.2)	71 (97.3)	0.003
Active immunization is a killed or weakened form of a disease-causing agent	49 (67.1)	65 (89.0)	0.001
Vaccination is for all ages	38 (52.1)	58 (79.5)	<0.001
Children get too many vaccines in the first two years of life	68 (93.2)	73 (100.0)	0.063
The immunization of the children should be started At birth	70 (95.9)	72 (98.6)	0.500
In some health situations, vaccines should not be given	43 (58.9)	60 (82.2)	<0.001
Vaccines can be given in combination	49 (67.1)	68 (93.2)	<0.001
If the child receives extra immunization, it is more effective and safer.	54 (74.0)	63 (86.3)	0.078
More than one dose of vaccine may be required for complete protection	50 (68.5)	70 (95.9)	<0.001

The parents’ overall pre-test and post-test scores were compared based on the number of questions answered correctly. The pre-assessments and post-assessments were completed by all of the 73 parents. The mean total knowledge score for the pre-test was 6.84 ± 1.52 and 9.15 ± 0.79 for the post-test, with a significant improvement of 2.31 points (*p* < 0.001, Table 3).

**Table 3:** **Total knowledge score before and after the intervention**

Scale	Mean	SD	Median	Minimum	Maximum	***P***value
**Knowledge**						<0.001
Pre	6.84	1.52	7.00	2.00	10.00	
Post	9.15	0.79	9.00	7.00	10.00	

In the baseline results there were no significant differences regarding knowledge about childhood immunization in subgroups pertaining to gender, age, marital status, number of preschool children, family size, race, religion, place of living, or employment status. However, there was a significant difference between parents’ knowledge and their education level and income (*p* < 0.001 and *p* = 0.005 respectively) (see Table 4).

**Table 4:** **Parents’ characteristics and baseline knowledge scores**

Characteristics	Knowledge scores		***P***value
	Mean	Median	
**Gender**			0.319
Male	7.11	8.00	
Female	6.80	7.00	
**Age**			0.663
20-30	6.63	7.00	
30-40	6.94	7.00	
>40	7.13	6.50	
**Marital Status**			0.869
Married	6.85	7.00	
Single	6.67	6.50	
**No. of Preschool Children**			0.720
1-2	6.85	7.00	
3-4	6.75	6.50	
**Family Size**			0.141
<4	6.86	7.00	
4-6	7.00	7.00	
>6	5.8	6.00	
**Race**			0.373
Malay	6.79	7.00	
Chinese	7.29	7.00	
**Religion**			0.373
Islam	6.79	7.00	
Buddhism	7.29	7.00	
**Place of Living**			0.373
Rural	7.29	8.00	
Urban	6.79	7.00	
**Employment Status**			0.166
Employed	6.95	7.00	
Unemployed	6.36	6.5	
**Education Level**			<0.001
Primary	4.67	5.00	
Secondary	6.33	6.00	
Tertiary Edu	7.53	7.00	
**Family Income**			0.005
< RM 1000	5.20	5.00	
RM 1001-2000	6.94	7.00	
RM 2001-3000	6.00	6.00	
RM 3001-4000	7.06	7.00	
> RM 4001	7.63	8.00	

### Parents’ questions and concerns

At the end of the seminar, some of the parents asked questions and shared their concerns about immunization. One mother asked, *“Why should I vaccinate my child against diseases that do not existe anymore? Where can my child get them from?”* She admitted that she stopped vaccinating her third and fourth child. A few parents also asked about the measles, mumps, and rubella (MMR) vaccine and if it really caused autism. A few parents admitted they did not vaccinate their children against measles, mumps, and rubella because they were afraid the vaccine might cause autism. Many parents also asked about the credibility of the information that they get from social media such as Facebook. One parent shared a story posted on Facebook about a child that got meningitis after an MMR vaccine and passed away. His parents believed it was due to the vaccine. The story made this parent change her mind about vaccinating her daughter. All the questions and concerns were answered and necessary explanations were given to the parents.

## Discussion

To our knowledge, this is the first study of its kind that used a short educational seminar to improve parents’ knowledge about immunization in Malaysia. This study demonstrated that providing a one-hour educational seminar to parents in a primary care clinic is an effective and practical strategy to improve parents’ knowledge about childhood immunization. However, the actual effectiveness of such interventions on immunization rates and status has not been studied.

Regarding demographic characteristics of the participants, mothers constituted the vast majority of the participants, indicating that child immunization is mainly under the mother’s responsibility, rather than the father’s. In this single group design study, a significant improvement in parents’ knowledge about immunizations was observed compared to baseline results, thus indicating that the one-hour educational seminar is an effective way to improve Malaysian parents’ knowledge about childhood immunization. It is well documented that parents’, and especially mothers’, knowledge has a great impact on the children’s immunization rate and maintaining up-to-date immunization status [7–13].

Our study identified some sociodemographic characteristics of the parents that were related to significant differences in immunization knowledge scores. Parents with a lower educational level or lower monthly income have lower overall knowledge about immunization compared with those who had better education and higher monthly income. This finding is consistent with previous studies [24, 27, 30–32]. There was no significant difference found between parents’ knowledge and other independent variables including, age, gender, marital status, number of preschool children, family size, race, religion, place of living, and employment status.

As health care providers are the main source of information for parents, it is important that they understand parents’ knowledge and familiarise themselves with the different demographic profile of their patients in order to remain updated about the issues of vaccine hesitancy [33, 34].

Educational interventions designed for parents can have important implications for improving vaccine uptake. Educating low-literate mothers by using pictorial messages and very simple language improved the completion rates of DPT-3/Hepatitis B vaccine by 39% [22]. In Germany, a study has showed that using balanced health information leaflets can increase girls’ and parents’ knowledge of the human papillomavirus vaccination and vaccination uptake [35]. A 20-minute educational presentation about human papillomavirus vaccination increased college females’ intent to vaccinate by nearly threefold [36]. Parents in Guatemala repeatedly revealed that workshops at the community level are the best way to increase their awareness and knowledge of vaccinations [37].

Our study results provide new data on parents’ knowledge and concerns about immunization. This information can enable policy makers to develop short, community-based, educational programmes at the clinics that provide vaccinations, especially for parents who have lower income and educational levels.

### Limitations

This study had some limitations: (1) a pre-post test for a single group without a follow up to determine the real effectiveness of the intervention on immunization rate and status and (2) whether the study succeeded to promote positive changes. These issues warrant further investigation in a longitudinal study. Furthermore, the study was conducted only with parents from Kuantan, the state capital of Pahang, and the findings may not to be extrapolated to the parents in the other states in Malaysia. However, the study generated data about parents’ knowledge of childhood immunization in Malaysia, thus providing baseline data to improve the current immunization rate and status. The study is not qualitative in nature. However, many questions were raised by parents post seminar when the platform was open for questions, and these questions were reported and included in the results due to their importance. The study findings may not reflect the knowledge of all Malaysian parents; rather, they reflect only the knowledge of those who actually participated in the programme. Therefore, the findings need to be interpreted within the context of study limitations.

## Conclusions

The educational intervention used in this study focused on improving parents’ knowledge about childhood immunization in Malaysia and has brought about a significant positive change in their knowledge about childhood immunization, compared with the baseline results. Further studies using a larger sample of parents from other states in Malaysia are required in order to assess the actual effectiveness of improving parents’ knowledge about childhood immunization and the immunization status of their children, and also to determine the cost-effectiveness of such an intervention.
